# Cognitive representation of “musical fractals”: Processing hierarchy and recursion in the auditory domain

**DOI:** 10.1016/j.cognition.2017.01.001

**Published:** 2017-04

**Authors:** Mauricio Dias Martins, Bruno Gingras, Estela Puig-Waldmueller, W. Tecumseh Fitch

**Affiliations:** a Berlin School of Mind and Brain, Humboldt University, Berlin, Germany; b Max Planck Institute for Human Cognitive and Brain Sciences, Leipzig, Germany; c Department of Cognitive Biology, University of Vienna, Vienna, Austria; d Institute of Psychology, University of Innsbruck, Austria

**Keywords:** Hierarchy, Recursion, Fractals, Music, Auditory

## Abstract

The human ability to process hierarchical structures has been a longstanding research topic. However, the nature of the cognitive machinery underlying this faculty remains controversial. Recursion, the ability to embed structures within structures of the same kind, has been proposed as a key component of our ability to parse and generate complex hierarchies. Here, we investigated the cognitive representation of both recursive and iterative processes in the auditory domain. The experiment used a two-alternative forced-choice paradigm: participants were exposed to three-step processes in which pure-tone sequences were built either through recursive or iterative processes, and had to choose the correct completion. Foils were constructed according to generative processes that did not match the previous steps. Both musicians and non-musicians were able to represent recursion in the auditory domain, although musicians performed better. We also observed that general ‘musical’ aptitudes played a role in both recursion and iteration, although the influence of musical training was somehow independent from melodic memory. Moreover, unlike iteration, recursion in audition was well correlated with its non-auditory (recursive) analogues in the visual and action sequencing domains. These results suggest that the cognitive machinery involved in establishing recursive representations is domain-general, even though this machinery requires access to information resulting from domain-specific processes.

## 1 Introduction

The capacity to represent and generate hierarchical structures is a fundamental human trait that is used in virtually every domain of activity. Even though this trait is to some extent present in other species (Bergman et al., 2003, Massen et al., 2014, Seyfarth and Cheney, 2008, Seyfarth et al., 2014), it seems to be especially developed in humans (Conway and Christiansen, 2001, Fitch and Friederici, 2012, Hauser et al., 2002, ten Cate and Okanoya, 2012, Vasconcelos, 2008). Not only are human-generated hierarchies more complex, but they are also more general, since the average person can generate visual, social, linguistic and action hierarchies (Altmann et al., 2003, Badre, 2008, Badre and D'Esposito, 2009, Badre et al., 2009, Bahlmann et al., 2008, Chomsky, 1995, Eglash, 1998, Fitch and Martins, 2014, Friederici, 2011, Hunyady, 2010, Jackendoff, 2003, Jackendoff, 2009, Kravitz et al., 2011, Picard et al., 2010, Zink et al., 2008). This complexity and generality could be explained either by a general increase in processing power, due to a larger brain, or by the existence of additional specialized processes or abilities in human cognitive architecture.

**Fig. 1:**
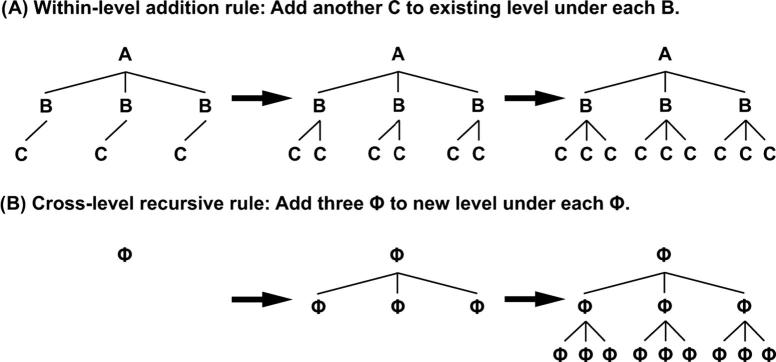
Recursive and non-recursive procedures to generate hierarchies. (A) Simple iterative procedures add elements to a hierarchy, within fixed levels. In order to generate a level under ‘C’, another rule would be required – add another ‘D’ under each ‘C’. (B) Recursive rules are more abstract. They can be used to characterize hierarchical relations across many different levels of the hierarchy. With the same rule (B), an infinite number of hierarchical levels could be added to the structure.

One such ability, which could potentially explain the human cognitive exceptionality, is recursion (Hauser et al., 2002). Recursion can be understood as the ability to embed elements within elements of the same kind (Fitch, 2010, Hulst, 2010, Martins, 2012). Recursion is a particular principle to represent and generate hierarchies which allows the generation of multiple levels with a single rule (Fig. 1B).

This increased generative power of recursion in comparison with other kinds of hierarchical principles is thought of as being instantiated by cognitive representations of a higher level of abstraction (Martins, 2012). For instance, instead of representing each hierarchical relation with its own rule, of the kind A → B and B → C (Fig.1A), humans are able to understand that these relations have commonalities, which allows the induction of a more general rule Φ → Φ (Fig.1B). To our knowledge, this level of hierarchical abstraction seems to be specifically available to human cognition (Berwick et al., 2013, Fitch et al., 2005, Hauser et al., 2002, ten Cate and Okanoya, 2012).

### 1.1 Cognitive assessment of recursion

Formally, structures that can be understood as recursive have been suggested to exist in visual art (Eglash, 1997), visuo-spatial processing (Martins, 2012, Martins and Fitch, 2012), music (Jackendoff and Lerdahl, 2006, Lerdahl and Jackendoff, 1996), architecture (Eglash, 1998), humour (Eisenberg, 2008), theory of mind (Miller, 2009, Tomasello, 2008), problem solving (Schiemenz, 2002), action sequencing (Pulvermüller & Fadiga, 2010), syntax (Chomsky, 1995, Karlsson, 2010, Mithun, 2010, Roeper, 2009), phonology (Hulst, 2010, Hunyady, 2010, Schreuder et al., 2009, Wagner, 2010), pragmatics (Levinson, 2013), conceptual structure (Hofstadter, 2000, Picard et al., 2010), mathematical proofs (Odifreddi, 1999), natural numbers (Hauser et al., 2002), and arithmetic operations (Friederici, Bahlmann, Friedrich, & Makuuchi, 2011). In all these domains it is possible to build recursive algorithms that generate hierarchical structures. However, it is not clear that in all these domains humans actually represent the recursive character of these structures, and use these representations productively. Such demonstrations require empirical rather than theoretical tools.

To our knowledge, the ability to induce recursive rules was empirically demonstrated first in the linguistic domain (Alegre and Gordon, 1996, Roeper, 2011). In this domain, recursion seems to be universally used (Reboul, 2012), and although some researchers argue that it is rare in common speech (Laury & Ono, 2010), most language users in the world are likely to have generated multiple recursive sentences in their lifetimes (for instance, compound noun phrases such as “[[[student] film]] committee]”). Furthermore, the ability to extract the correct meaning from recursive sentences seems to be available early during ontogeny (Alegre and Gordon, 1996, Roeper, 2009). This interesting relationship between language and recursion, yet undemonstrated in other domains, has led some authors to propose that recursion is a domain-specific “linguistic computational system […], independent of the other systems with which it interacts and interfaces” (Fitch et al., 2005, Hauser et al., 2002). This hypothesis implies that the use of recursion in other domains is dependent on verbal resources. Coincidentally, the ability to perform second-order theory of mind tasks (e.g., [I think that [she thinks that [John thinks something]]]) correlates with language abilities (Miller, 2009, for a review), and verbal interference tasks block the ability to use natural numbers (Gordon, 2004). These results were taken as strong evidence that recursion is a linguistic domain-specific ability.

Recently, in a series of experiments, human adults and children have also been shown to represent recursion in the visuo-spatial domain (Martins et al., 2014, Martins et al., 2014, Martins et al., 2015). In this domain, subjects were able to induce recursive rules generating visual fractals, and to use these rules productively. Crucially, this ability was not specifically related with grammar comprehension (Martins, Laaha, et al., 2014), and it neither required verbal resources (Martins et al., 2015), nor generated activation in classical language brain areas (Martins, Fischmeister, et al., 2014). However, performance correlated with an action sequencing task, the Tower of Hanoi, which is best solved using recursive strategies (Martins, Fischmeister, et al., 2014).

These findings suggest that recursion does not necessarily require linguistic resources (arguing against the primacy of language). However, it is still possible that the same cognitive machinery is used to implement recursion in both domains (language and vision), if a domain-general (abstract) code were used instead of a linguistic or verbal one (Martins et al., 2015).

In this paper, we aim to expand our understanding of recursion in human cognition by focusing on another non-linguistic domain – using acoustic stimuli – and measure how recursive capacities in this domain correlate with recursion in other domains. Towards this goal, we will assess how humans represent “musical fractals”.

### 1.2 Hierarchical processing of music

Like language, music is a domain known to require the processing of hierarchical relations (Jackendoff, 2009, Jackendoff and Lerdahl, 2006, Koelsch et al., 2013, Rohrmeier, 2011). These relations involve the embedding of discrete acoustic events into higher-order structures, according to their rhythmical and pitch relationships. For instance, in tonal structures, there are precise relations between tones and the context in which these tones are embedded. Thus, in Western music, the same tone (or chord) can be perceived as decreasing or increasing the tension of a musical sequence according to the context, creating emotional feelings of completeness versus incompleteness (Jackendoff & Lerdahl, 2006). The rules that govern these dynamics of tension and attraction are usually acquired during the process of enculturation with the musical canon (Tillmann, 2012, Tillmann and Bigand, 2004), and are commonly referred to as musical syntax, in analogy to linguistic rules (e.g., Fadiga et al., 2009, Jeon, 2014, Koelsch, 2006, Koelsch et al., 2013, Patel, 2003, Patel et al., 1998, Sammler et al., 2009).

But unlike in language, the understanding of long-distance hierarchical relations in music seems to be particularly difficult, especially for non-musicians (Besson et al., 1994, Tillmann and Bigand, 2004). The effect of expertise seems to be more influential in the ability to make explicit decisions, rather than in perceptual aspects (Besson & Faïta, 1995)

To our knowledge, only one study has suggested that non-musicians might be sensitive to long-distance relations in music (Koelsch et al., 2013). However, since many other studies have failed to detect this sensitivity (e.g., Cook, 1987, Tillmann et al., 1988), it remains unclear to which extent non-musicians can represent complex hierarchical relations in music, even though some capacity can be acquired through passive listening (Bigand & Poulin-Charronnat, 2006). Interestingly, some studies suggest that the processing of syntactic violations in music seems to elicit neural signals analogous to the processing of linguistic syntax (Koelsch, 2006, Maess et al., 2001, Patel et al., 1998, Sammler et al., 2009). This provides evidence for an influential view that music and linguistic syntax have common resources, and perhaps a common evolutionary history (Brown, 2000, Fitch, 2006, Patel, 2003).

Finally, hierarchical relations of tonal structures have also been formalized as recursive (Jackendoff and Lerdahl, 2006, Lerdahl and Jackendoff, 1983, Rohrmeier, 2011). However, as in many other domains, it remains an open question whether human adults, and especially non-musicians, can induce these recursive rules and use them productively. In this article we describe an attempt to experimentally answer this question.

### 1.3 Aims

The current study has two main aims: The first is to assess whether human adults, in particular non-musicians, are able to represent hierarchical relations in the auditory domain. In particular, we will test whether they are able to induce and apply within-level iterative rules (Fig.1A) and cross-level recursive rules (Fig.1B) in the domain of structured tonal sequences. The second goal is to assess whether the ability to represent recursion in the musical domain correlates with analogous tasks in the visual and action domain. This will address the question of whether the ability to form recursive representations relies on domain-specific resources or a domain-general cognitive machinery.

## 2 Experiment 1: Can humans represent recursion in the auditory domain?

Previous research by our group has shown that the ability to represent recursion is present in the general population in the visuo-spatial domain (Martins, 2012; Martins, Fischmeister, et al., 2014; Martins, Laaha, et al., 2014; Martins et al., 2015). In the current experiment, our goal was to determine whether this ability is also present in the auditory domain. Analogous to our work in vision, we applied recursive rules over sequences of tones, and tested whether participants were able to extract these rules. We employed a two-alternative forced-choice paradigm: participants were exposed to three steps of a recursive process generating auditory fractals (using pure tone sequences), and then asked to discriminate between a well-formed fourth step of the same process and a foil. In addition to this task, which we named Auditory Recursion Task (ART), we devised a control task, the Auditory Iteration Task (AIT), which shared the hierarchical, iterative and sequential aspects of ART, but which did not use recursive procedures to generate the hierarchical structures. Our previous research - behavioral, developmental and neuroimaging - suggests that hierarchical processing taps into different systems depending on whether an iterative or recursive rule is primed (Martins, 2012; Martins, Fischmeister, et al., 2014; Martins, Laaha, et al., 2014).

In this experiment, we tested both musicians and non-musicians. On the one hand, musicians are known to be able to represent rules governing tone structures (Tillmann, 2012, Tillmann and Bigand, 2004, for reviews). If the ability to represent recursion is available in the auditory domain, then musicians should be able to adequately encode our tone sequences as formed by recursive rules. On the other hand, non-musicians are known to have difficulties in explicitly representing some elementary qualities of musical sounds (Rammsayer and Altenmüller, 2006, Tervaniemi et al., 2005). Even though non-musicians might be able to process the dynamics of musical tension and relaxation resulting from hierarchical relations (Koelsch et al., 2013, Maess et al., 2001), their ability to process pitch and rhythm is inferior to that of musicians (Rammsayer and Altenmüller, 2006, Tervaniemi et al., 2005, Tillmann and Bigand, 2004), as is their ability to retain auditory sequences in memory (Cohen, Evans, Horowitz, & Wolfe, 2011). In our task, we used somewhat impoverished stimuli from a musical viewpoint. Although we used rhythmical and tone structures, these comprised only simple pitch intervals and durational ratios. Our goal was to increase the likelihood that non-musicians would be able to parse our stimuli as structured sequences of tones. Nevertheless, a failure of the non-musician population could either mean that the ability to represent recursion is not available, or that non-musicians cannot easily parse tone structure in general. General impairments could be caused by bottlenecks either at the proximal auditory level (Bigand & Poulin-Charronnat, 2006), or at the memory level (Cohen et al., 2011), since music stimuli are fast and transient. We tried to reduce the latter effect by allowing participants to listen to all stimuli as many times as they wished before providing a response.

Finally, we also included an initial training task, in which stimuli were simple structures of three notes, directly utilizing the ability to parse tone contour.

In sum, our goal was to assess whether humans could represent recursive rules in auditory sequences. If this ability is potentially present in human cognition, then at least musicians should perform adequately. To test whether this capacity was present in the non-expert general population, we also tested non-musicians.

### 2.1 Methods

#### 2.1.1 Participants

We tested 30 non-musicians (17 females) aged between 19 and 38 (*M* = 25.1, *SD* = 5) with the Auditory Recursion Task, and 24 different non-musicians (17 Females) aged between 19 and 46 (*M* = 25.7, *SD* = 7) with the Auditory Iteration Task. In addition, we tested 20 musicians (16 females) aged between 19 and 38 (*M* = 24.0, *SD* = 4) with the Auditory Recursion Task. Musicians were defined as participants with more than 8 years of musical training who regularly practiced an instrument or singing (more than once a week). Non-musicians were participants with less than 2 years of music classes who reported not regularly playing any musical instrument. All participants were recruited at the University of Vienna. All were tested using the same experimental apparatus, and all reported normal or corrected-to-normal visual and auditory acuity. All participants gave their prior written consent, and were paid 10 euros. The research conformed to institutional guidelines of the University of Vienna and Austrian national legislation regarding ethics.

#### 2.1.2 Stimuli and procedure

**Fig. 2:**
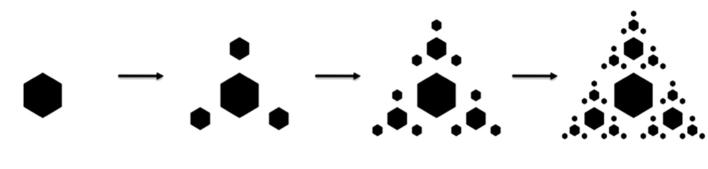
Example of a recursive process generating a visual fractal.

**Stimulus generation.** The stimuli comprising the Auditory Recursion Task were built as an auditory analog of visual fractals (Mandelbrot, 1977). Visual fractals are structures that can be generated from single constituents such as squares or triangles (*the initiators*) by applying a simple transformation rule (*the generator*) a given number of times (iterations). The structures generated by iterating this process are hierarchical and self-similar (see Fig. 2 for a exemplary overview of such a process).

In the visual domain, hierarchical level is denoted by constituent size (larger constituents being dominant, and smaller subordinate), and the transformation rule (*generator*) encodes the spatial position of a set of subordinate elements (three surrounding elements in Fig. 2) in relation to the dominant. Here, we built our auditory fractals using auditory features analogous to these parameters: we took note duration and pitch to denote hierarchical level (longer and lower-pitched notes being dominant over shorter and higher ones); and tone space as the parameter being modulated by the *generator*. Specifically, for each tone present in one iteration (hierarchically dominant), we added three new tones (hierarchically subordinate) higher in pitch (and shorter in duration), with a particular contour (variably ascending or descending), and at a certain pitch distance from the dominant (inter-level interval); these subordinate-note contours constitute the recursive rule operative over multiple hierarchical levels.

**Fig. 3:**
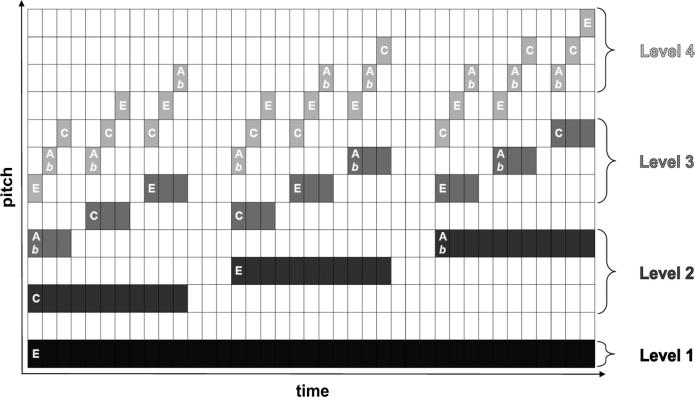
Example of a tonal auditory fractal. This auditory structure is composed of 4 hierarchical levels, denoted with different shades of gray. The dominant level (Level 1) is a low-pitch note with duration of 7.3 s. The second level (Level 2) is composed of three notes, each with a duration of slightly less than 1/3 the dominant note (Level 1), and with short silent pauses between them. This set of three notes is arranged in an ascending directionality, with a fixed pitch interval between each pair within these three notes. The same principle is used to generate Level 3, in which each set of three notes is added at a certain pitch interval in relation to a dominant note (each note in Level 2).

**Fig. 4:**
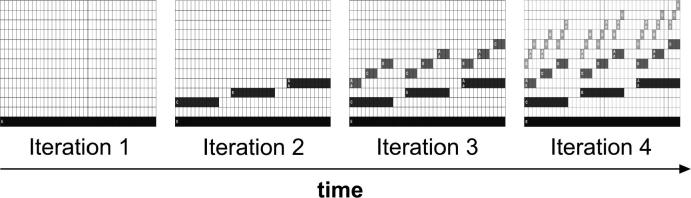
Recursive process generating a tonal auditory fractal. At each step of the recursive process, a new hierarchical level is added (in the figure, a lighter shade of gray) containing notes of a higher pitch and shorter duration. A certain note is dominant (e.g., Level 1) over a set of three notes (e.g., Level 2) if they occur simultaneously in the sequence (time-wise), and the former is of a lower pitch and longer duration. The pitch and rhythmical relations between dominant and subordinate elements are kept constant recursively across hierarchical levels. Thus, if the dominant level displays an ’ascending’ contour, so does the subordinate level. This between-level regularity occurs regarding the distance between levels, and distance within level (between notes of each set of three). In our recursion task, participants were first exposed to the first three iterations of the process, and then asked to discriminate between the fourth iteration and a foil (Fig. 5).

A typical example of a music fractal is represented in Fig 3. The target stimulus was built in four steps or iterations (Fig. 4). The first iteration stimulus consists simply of a low-pitch pure tone with a duration of 7.3 s (*the initiator*). The second iteration retains the initial tone, and adds a set of three new tones, according to a particular rule (*the generator*). This rule manipulates: (i) pitch contour, which can be either ‘ascending’ or ‘descending’; (ii) pitch interval between consecutive tones, which can correspond either to four semitones (major third) or eight semitones (minor sixth); and (iii) pitch interval between successive levels, which can also correspond to either four or eight semitones (Supplementary Materials I contain sound files corresponding to the four successive steps involved in the generation of a tonal auditory fractal). The same *generator* was applied to build all new hierarchical levels. Thus, the pitch and rhythmical relations between dominant and subordinate elements were kept constant recursively across hierarchical levels. This produced a hierarchical self-similar structure.

These pitch intervals (major third and minor sixth) were chosen because they are consonant, and because simultaneous combinations of major thirds and minor sixths also usually generate consonant combinations (with the exception of augmented chords). Moreover, an interval of 4 semitones is considered sufficient to ensure auditory stream segregation between the various hierarchical levels (Vliegen & Oxenham, 1999). Furthermore, to maximize auditory stream segregation, we applied different amplitude modulation rates to the tones at different levels, with no amplitude modulation (0 Hz) for the lowest (first) and highest (last) hierarchical levels and amplitude modulation rates of 6 and 18 Hz for the second and third hierarchical levels (Grimault, Bacon, & Micheyl, 2002). The pure tone frequencies used in the stimuli ranged between 50.5 Hz and 3846 Hz, a frequency range within which humans can reliably recognize frequency intervals (Semal & Demany, 1990). For comparison, the note frequencies found on a 88-key modern piano range between 27.5 and 4186 Hz.

**Fig. 5:**
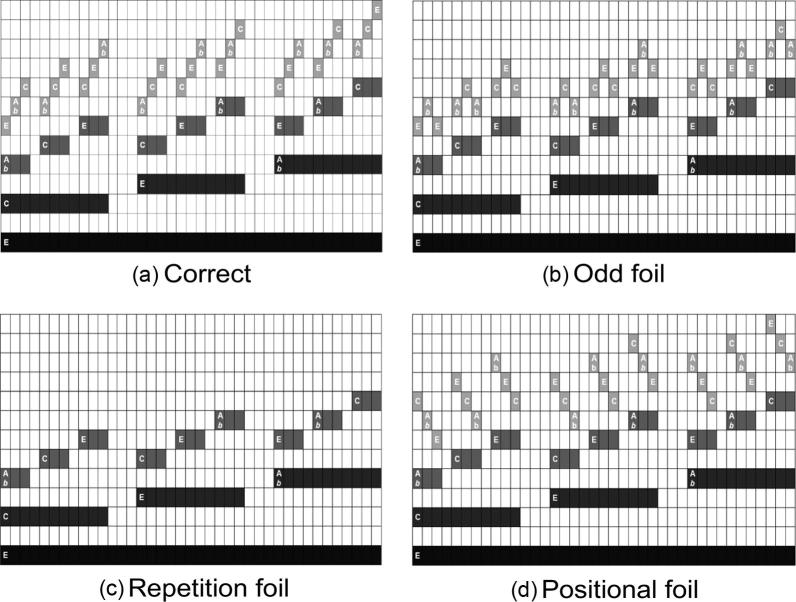
Foil categories. For each music fractal, we generated the correct continuation (a.) of the recursive process in Fig. 4 and three different foils. The ‘Repetition’ foil (c.) is identical to the third iteration, and fails to continue the process, however it keeps the overall configuration of the auditory sequence (‘ascending’) constant. In both ‘Odd’ (b.) and ‘Positional’ (d.) foils, a new hierarchical level is added to the structure, however, this level is not consistent in contour with the pattern in previous iterations. In ‘Odd’ (b.) foils, the last note of each set of three is of the same pitch as the first note within that set. This disrupts the expected directionality (ascending or descending). In ‘Positional’ foils (d.), the directionality is consistent within each set of three, but not consistent with other hierarchical levels. In this example, the higher level has a descending contour, even though the previous levels display an ascending contour.

We produced four successive iterations of 24 different types of auditory fractals, generated using custom code in MATLAB R2012b (Mathworks, Natick, MA). For each of these 24 fractals, we produced (1) a correct fourth continuation of the first three iterative steps and (2) an incorrect continuation as a “foil” stimulus. This incorrect fourth iteration was produced by applying a different *generator* to the third stage. There were three categories of foils (Fig. 5): (i) positional, (ii) odd, and (iii) repetition (sound files corresponding to well-formed fourth iterations and foils can be found in Supplementary Materials II).

**Auditory Recursion Task (ART).** Participants sat in front of a computer screen and used the mouse to interact with a custom GUI interface implemented using Python programming language. Sounds were delivered through Sennheiser HC 520 headphones.

The task was composed of 24 trials. The set of 24 stimuli was a fully balanced distribution of all categories (see stimulus generation): inter-note interval (with 12 stimuli using the major third and 12 the minor sixth), direction (12 ascending and 12 descending), inter-level interval (12 using the major third and 12 the minor sixth), and foil (8 repetition, 8 position, and 8 odd).

**Fig. 6:**
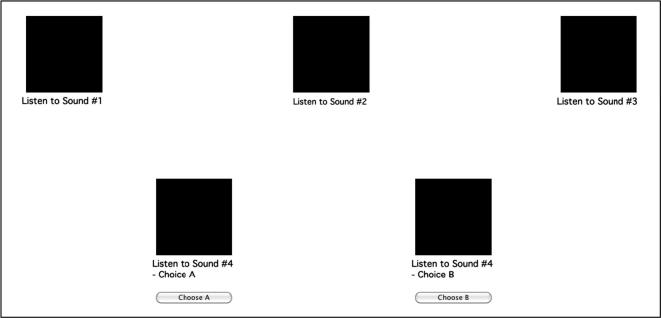
Screenshot of a typical MRT trial. At the beginning of teach trial, sounds (iterations) #1, #2 and #3 were automatically played in sequence. Participants were then asked to choose from 2 alternatives (Choice A and Choice B) which corresponded to the correct Sound #4. Participants could use the mouse to click on the black squares in order to listen to the corresponding sounds. Participants could listen to the stimuli as many times as they wished. Responses were presented by clicking on the buttons ‘Choose A’ or ‘Choose B’. These buttons became active only after all 5 sounds had been played at least once. After a choice was presented, the next trial started.

On each trial, a set of five plain black square buttons was displayed on the screen, one button corresponding to each different tone sequence (Fig. 6). The squares corresponding to the iterations 1, 2 and 3 were depicted on the top of the screen; and the possible continuations (correct and foil) were displayed at the bottom. The position (LEFT and RIGHT) of the correct and incorrect fourth iterations were counterbalanced.

At the beginning of each trial, iterations 1, 2, and 3 were automatically played in sequence. Each sound had a duration of 7.3 s, and they were presented with a pause of 1 s between them. There was a visual cue (change from black to gray) indicating which iteration (square button) was being played. After the 3 first iterations were played, participants were asked to choose from 2 alternatives (Choice A and Choice B) which corresponded to the correct fourth iteration. Participants could listen to the alternatives (and repeat the other sounds) as many times as they wished by clicking on the corresponding squares. Responses were delivered by clicking on the buttons ‘Choose A’ or ‘Choose B’ displayed under the corresponding black squares (Fig. 6). These buttons became active only after all 5 sounds were played. After a choice was made, the next trial started.

Since participants were allowed to listen to all sounds as many times as they wished, the total duration of the task was variable. The average was around 45 min.

**Fig. 7:**
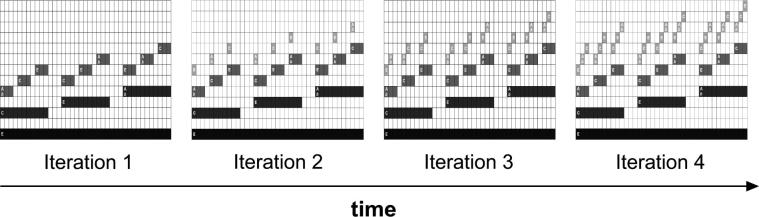
(Non-recursive) iterative process generating a music fractal. Each iteration of this process adds one subordinate note (Level 4) for each dominant note (Level 3). The pitch interval between the three notes being added to Level 4 (within the duration of each note in Level 3) is kept constant. To solve this task, participants need to discriminate the pitch contour (ascending or descending) between the first two light gray notes (within each set of three) in Iteration 3, and generalize this contour to Iteration 4. Foil categories were similar to ART.

**Auditory Iteration Task (AIT).** The experimental procedure and stimuli used in AIT were similar to what was described above for ART. The crucial difference between the tasks was the procedure used to generate Iteration 4 (Fig. 7). While in ART each iteration embedded the new tonal elements recursively within new hierarchical levels (different tone durations at each step, Fig. 4), in AIT elements were embedded within a single fixed hierarchical level (always the same tone duration), without adding new levels to the structure (sound files corresponding to AIT sequences and foils can be found in Supplementary Materials III). Experimentally, this means that Sounds 1, 2 and 3 were different between ART and AIT. However, the choice pairs (Choice A and Choice B) were the same between tasks. Foil stimuli were also identical, except for the ‘Repetition’ foils, since Iteration 3 in AIT is different from Iteration 3 in ART. All other parameters were kept constant between tasks.

**Training.** The goal of the training procedure was to allow participants to familiarize themselves with the overall framework, and with the concept of ascending and descending sequences of three tones, a crucial prerequisite to perform both ART and AIT. Each trial presented participants with a sequence of two tones (Sound #1), in which the second tone was either of a higher or lower pitch than the first one (ascending and descending direction, respectively).

After this sequence was played, we asked participants to decide, from a set of two alternatives (Choice A and Choice B), which one correctly completed ‘Sound #1’. The correct item was a sequence of three tones in which the third followed the same direction (ascending or descending) as the first two. Foils and interface were similar to ART, except that only one square was presented in the top center of the screen (corresponding to Sound #1).

#### 2.1.3 Procedure

All participants started the procedure by filling in a questionnaire concerning their level of musical education and practice. All performed a training session as described above. Then, depending on the group they were assigned to, each participant either performed the ART or AIT. For both these tasks, instructions were minimal. Participants were simply asked to listen carefully to the first 3 iterations and try to imagine how the fourth iteration would sound. They were then asked to select the correct fourth iteration among 2 alternatives (A complete PowerPoint presentation with instructions can be found in Supplementary Materials IV). Importantly, participants were not explicitly instructed on the concepts of recursion or iteration, and had to extract these regularities by themselves while being exposed to stimuli examples. The overall procedure lasted approximately 45 min.

#### 2.1.4 Analysis

To compare the proportion of correct answers across groups and conditions, we used generalized linear mixed model analysis which was implemented in R 3.1.1 (R Core Team, 2015) using the glmer function from package lme4 to build the models (Bates, Maechler, Bolker, & Walker, 2015), the fitLMER function from package LMERConvenienceFunctions to select the best-fitting models (Tremblay & Ransijn, 2015) and the anova function from package car to obtain significance tests (Fox and Weisberg, 2011). Correlational analyses were done using Spearman’s rho. To assess whether there were effects of learning, we tested whether accuracy and RT across trial were well explained by power curves (Anderson, 1982). To this effect we performed Curve Estimation analyses using SPSS 22 (IBM).

### 2.2 Results

#### 2.2.1 Training

Prior to both ART and AIT, participants performed a simple training in which they had to discriminate the correct continuation of a (either ascending or descending) sequence of two tones (separated by either 4 or 8 semi-tones). Mean accuracy in the training was 77% in the group of musicians (*SD* = 24), and 76% in the group of non-musicians (*SD* = 17). The best fitting model included effects of ‘foil’ (Positional: *b* = 1.0, *SE* = 0.16; Repetition: *b* = 0.4, *SE* = 0.15, χ^2^(2) = 41.9, *p* < 0.001), ‘tone distance’ (major third: *b* = 0.5, SE = 0.16, χ^2^(1) = 15.7, *p* < 0.001) and ‘directionality’ (descending: *b* = −0.5, *SE* = 0.13, χ^2^(1) = 18.7, *p* < 0.001). Having musical training did not significantly affected accuracy in the training (*b* = 0.3, *SE* = 0.4, χ^2^ (1)= 0.7, *p* = 0.4).

#### 2.2.2 ART vs AIT: non-musicians

**Fig. 8:**
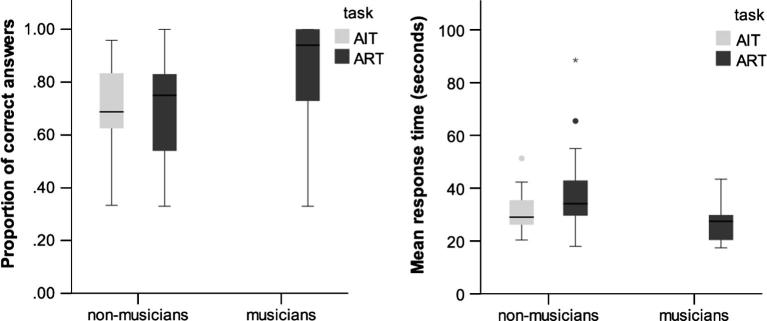
Accuracy (left) and response times (right) in ART. The boxplot divides the scores into quartiles, the ‘box’ represents the distance from the 25th percentile to the 75th percentile (interquartile range). The horizontal dark line is the median. ° are outliers deviating from the box between 1.5 and 3 times the interquartile range; * are outliers deviating from the box more than 3 times the interquartile range.

On average, participants scored 70.8% correct (*SD* = 18) in ART and 70.1% in AIT (*SD* = 16%) (Fig. 8). Response accuracy was above chance in both tasks (binomial tests, *p* < 0.001). Our best accuracy model converged to an overall effect of foil category (χ^2^(2) = 7.7, *p* = 0.02). We will probe into this effect in the “Rejection of foils section”. Importantly, we found no accuracy differences between ART and AIT (*b* = −0.07, *SE* = 0.25, χ^2^(1) = 0.1, *p* = 0.8), and no effects related to other stimuli features (inter-tone, inter-level interval, and direction) (all *p*s > 0.1).

Mean RT, measuring from the end of the last obligatory playback, was 37.5 s (*SD* = 10.7) in ART and 31.1 s (*SD* = 6.1) in AIT. The best-fit model also included effects of foil category (χ^2^(2) = 10.1, *p* < 0.01). There were no significant differences between tasks (*b* = −3.8, *SE* = 2.4, χ^2^(1) = 2.5, *p* = 0.1), and no other effects related to stimuli features (all *p*s > 0.1).

#### 2.2.3 Learning curves

In order to assess whether participants induced recursive and iterative rules, we analyzed performance across trials (1–24) and including all foil categories (repetition, positional and odd).

**Fig. 9:**
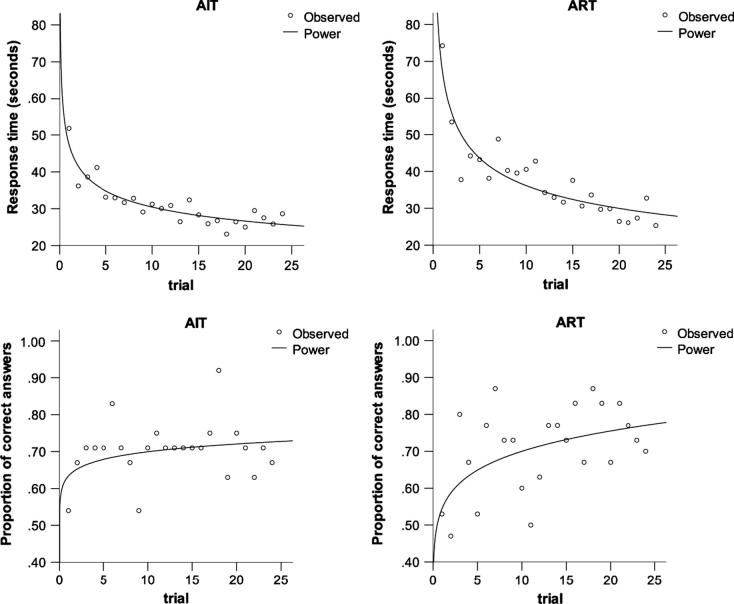
Response time (top) and response accuracy (bottom) learning curves. Auditory Iteration Task (AIT; left); Auditory Recursion Task (ART; right). In this figure we have included only non-musicians, since performance was at ceiling for most musician participants.

In ART, participants’ proportion of correct answers increased with trial number, significantly fitting a power curve (*F*(1,22) = 8.0, *p* = 0.01, *R*^2^ = 0.27, and suggesting a learning effect (Fig. 9). In AIT, this effect was not significant (*F*(1,22) = 2.5, *p* = 0.1, *R*^2^ = 0.10). When we analyzed the evolution of RT across trials, we found that in both tasks RT became significantly shorter in later trials, also fitting a power curve (*p* < 0.001, *R*^2^ = 0.80, for both tasks).

In the musicians group, we found similar learning effects for both accuracy (*F*(1,22) = 7.0, *p* = 0.015, *R*^2^ = 0.24) and RT (*F*(1,22) = 177, *p* < 0.001, *R*^2^ = 0.89) in ART.

#### 2.2.4 Rejection of foils

**Fig. 10:**
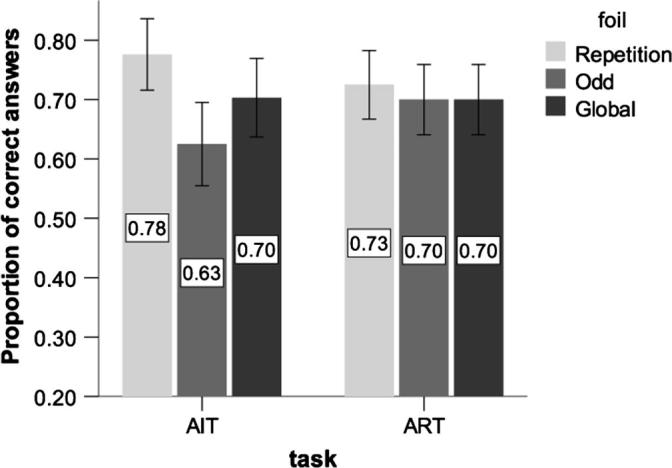
Response accuracy across foil categories (non-musicians only). AIT (Auditory Iteration Task); ART (Auditory Recursion Task). In this analysis we have included only non-musicians.

In ART, the best fit was an intercept model only (null model). Participants were equally likely to correctly reject the incorrect choice across all foil categories (Fig. 10) (χ^2^(2) = 0.5, *p* = 0.8). In contrast, in AIT, we found that the best model included an effect of foil category (odd: *b* = −0.38, *SE* = 0.22; repetition*: b* = 0.4, *SE* = 0.24, χ^2^ (2) = 11.4, *p* < 0.01): participants were more likely to reject ‘repetition foils’ than ‘odd’ foils (*p* = 0.03) (Fig. 10). For both tasks, we found that no other factor, such as directionality (ascending vs. descending), inter-note interval (4 vs. 8 semitones) and inter-level interval (4 vs. 8 semitones) was a significant predictor of accuracy in either of the tasks (all *p*s *>* 0.1).

#### 2.2.5 ART: Musicians vs. non-musicians

On average, the percentage of correct answers in ART was 84% (*SD* = 37) in the group of musicians and 70.8% (*SD* = 18) in the group of non-musicians (Fig.8). The best-fitting model included a significant main effect of music training (*b* = 2.1, SE = 0.5, χ^2^(1) = 8.4, *p* < 0.001) and a significant interaction between foil and music training. In particular, musicians were worse in rejecting ‘Repetition’ foils than other kinds of foils (*b* = −2.0, SE = 0.4, χ^2^ (2) = 32.6, *p* < 0.001). There were no other significant stimuli effects (inter-tone, inter-level interval, and direction) (all *p*s > 0.1).

Mean RT was 26.8 s (*SD* = 20) for musicians and 37.5 s (*SD* = 10.7) for non-musicians. The best model included a main effect of music training (*b* = 10.1, SE = 1.6, χ^2^ (1) = 14, *p* < 0.001), and no other significant terms (all *p*s > 0.1).

### 2.3 Discussion

In this experiment we found that participants, both with and without musical training, are able to induce recursive rules governing the generation of music fractals, and to use these rules productively. We have shown that, without any feedback or explicit instructions, participants were able to learn and use recursive rules in the Auditory Recursion Task (ART). They were able to induce and transfer information from one trial to the next, as shown by the learning curves, and to consistently reject incorrect continuations of recursive processes across three different foil categories. This suggests that no single simple auditory heuristic was used to solve the task.

We also introduced a control task, the Auditory Iteration Task (AIT), which is similar in many respects to ART. In particular, participants were asked to discriminate between similar pairs of choices (correct and foil) in AIT and ART, but the type of rules that were used to generate these choice items was different. In ART the generating rule is recursive, and each step generates a new level hierarchical level, while in AIT the generating rule is iterative, and performs transformations within a fixed level of the hierarchy.

When we compared performance across the two tasks, we found that accuracy was globally similar: participants performed adequately in both AIT and ART. However, we also found differences between the tasks: (1) the accuracy learning curve was somewhat steeper in ART than in AIT; (2) While participants rejected all foil categories equally well in ART, they were particularly bad at rejecting the ‘Odd’ foils in AIT.

These differences all align well with previous findings in the visual domain (Martins, 2012; Martins, Laaha, et al., 2014). The strategy used to acquire the recursive principles might differ from simple iteration (Martins, 2012). This strategy involves the induction of a rule, which seems to play a bigger role in ART than in AIT, as suggested by the accuracy learning curve (Dewar & Xu, 2010). The principle induced in ART allows participants to reject equally well all three different kinds of foils. In AIT, however, performance was less consistent across foil categories, suggesting that heuristic strategies might play a bigger role, although not an exclusive one, in solving this task. Partial heuristics are adequate to reject ‘Repetition’ foils (in which the foil is identical to the third iteration), but inadequate to reject ‘Odd’ foils, which are somewhat more subtle. The difficulty in rejecting ‘Odd’ foils while using iterative, but not recursive, representations of hierarchical structures is also present in the visual domain (Martins, Laaha, et al., 2014) and might be caused by a greater ability to process fine-grained details of hierarchical structures when using more abstract representations (Alvarez, 2011). We suggest that this is the case of recursion vs. iteration, as suggested by our previous behavioral, developmental and neuroimaging findings (Martins, 2012; Martins, Fischmeister, et al., 2014; Martins, Laaha, et al., 2014).

#### 2.3.1 Musicians vs. non-musicians

Although non-musicians performed above chance in ART, their performance was not very high (71%), at least in comparison with the ability to perform an analogous task in the visuo-spatial domain (>84%). Interestingly, the accuracy level of musicians (84%) was similar to the accuracy of the general population in the visuo-spatial recursion task (Martins, 2012). This finding is interesting and suggests, in line with previous findings, that specific practice and expertise effects could play a bigger role in the auditory domain than in the visual domain (Cohen et al., 2011). Even though the capacity to process hierarchies in both domains may be cognitively correlated, the processing of local (vs. global) features in the auditory domain (e.g., ‘direction’ of tone sequences) seems to be more difficult than the processing of local features in the visual domain (e.g., geometric shapes) (Bouvet, Rousset, Valdois, & Donnadieu, 2011). In that regard, musicians generally show smaller pitch interval discrimination thresholds than non-musicians, although the thresholds previously reported for non-musicians were also under 4 semitones, which was the smallest inter-note or inter-level interval used here (McDermott, Keebler, Micheyl, & Oxenham, 2010). Alternatively, these differences in difficulty might be related not to the domain itself (visual vs. auditory), but simply reflect the fact that, as a task, ART is more difficult than VRT. Perhaps if VRT were made more difficult it would similarly depend on practice effects. Future research investigating the capacity of visually sophisticated experts on a difficult version of VRT could address this question.

In experiment 2 we compare ART and AIT with their analog in the visual domain, and test our participants with specific melodic memory tasks. With this procedure we will assess how much of ART performance is explained by recursion itself, and how much is explained by general auditory or musical processing.

## 3 Experiment 2: Is auditory recursion domain-specific?

In Experiment 1, we have shown that human adults can perform adequately in an auditory recursion task and in an auditory iteration task. We have also shown that participants’ behavior was consistent with rule induction, especially in the Auditory Recursion Task (ART), given that there was consistent performance across items using different foil categories, and a consistent learning effect.

In this experiment we will attempt to investigate more closely what kind of rule is being induced in ART. To that effect, we will investigate the relationship between accuracy in ART and in other non-auditory recursive tasks, namely the Visual Recursion Task (VRT) (Martins et al., 2015) and the Tower of Hanoi task (ToH; which requires recursive action sequencing) (Goel and Grafman, 1995a, Halford et al., 1998, Halford et al., 2010). Performance in VRT and ToH was previously shown to be correlated (Martins, Fischmeister, et al., 2014) and they thus offer appropriate tools to tap into the ability to establish cognitive representations of recursion. A close correlation between ART, VRT and ToH would corroborate that ART taps into something specific to recursion.

However, ART is an auditory task which also requires the ability to perceive musical tone structure. To quantify these general musical and auditory expertise effects we included in the test battery our control Auditory Iteration Task, a Melodic Memory Task (MMT), which requires the ability to compare transposed tone contours (Mullensiefen, Gingras, Musil, & Stewart, 2014), and a (non-auditory) Visual Iteration Task (VIT), to control for variance explained by specific visual resources. In addition, we took into account the number of years of musical training of the participants.

In sum, with this test battery, comprising ART, AIT, ToH, VRT, VIT, MMT, combined with the number of years of musical training, we hoped to investigate whether, after controlling for effects specific to auditory and visual processing, ART was specifically correlated with other recursive tasks. If this were the case, this would support the hypothesis that our novel task taps into the ability to represent recursion in the auditory domain, and would be relevant to the question of whether recursion is domain-general or domain-specific. The general methodology for this investigation involved exploratory correlations between task scores and a Principal Component Analysis.

### 3.1 Methods

#### 3.1.1 Participants

We tested 40 participants (24 females) aged between 18 and 49 years (*M* = 26, *SD* = 7). In contrast to Experiment 1, in this experiment we included participants with various levels of musical expertise, from no musical training to 16 years of musical training (*M* = 3.3, *SD* = 4). All participants were tested with all tasks and were paid 20 euros. All other admission and testing conditions were similar to experiment 1.

#### 3.1.2 Auditory recursion and auditory iteration tasks

We used exactly the same ART and AIT versions as in Experiment 1.

#### 3.1.3 Visual recursion and visual iteration tasks

**Fig. 11:**
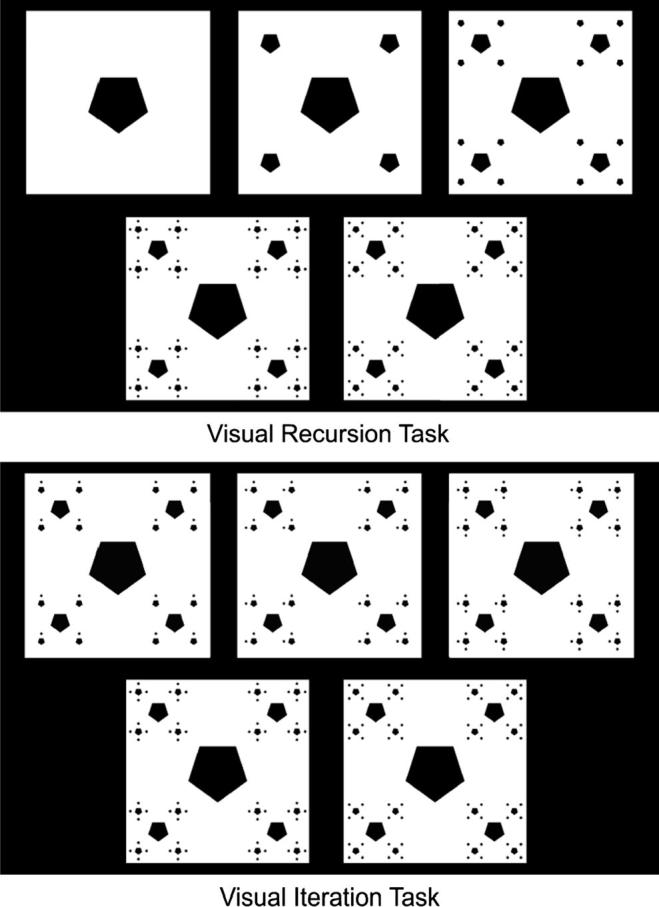
Screenshots of the Visual Recursion Task (top) and the Visual Iteration task (VIT).

The Visual Recursion Task (VRT) and Visual Iteration Task (VIT) were adaptations of the tasks used and described in detail elsewhere (Martins, 2012; Martins, Fischmeister, et al., 2014; Martins, Laaha, et al., 2014). These were visual analogs of ART and AIT. In both tasks, participants were exposed to a sequence of three images (Iterations 1, 2, and 3) which depicted a certain process generating a fractal. This process could be iterative or recursive (Fig. 11). After being exposed to the first three iterations, participants were asked to discriminate, from two choices, the image corresponding to the correct continuation of the previous sequence of three (i.e. the fourth iteration). One of the choices was the correct image, and the other was a foil. Foil categories were identical to the ones used in ART and AIT (Odd, Positional, and Repetition).

Both VRT and VIT were composed of 27 trials, 9 of each foil category. Variability was achieved by varying the number of constituents composing the visual fractal, as described in (Martins, Laaha, et al., 2014).

#### 3.1.4 Tower of Hanoi (ToH)

ToH is a visuo-motor task widely used in clinical and cognitive assessment (Shallice, 1982). ToH requires the hierarchical movement of disks across pegs to solve puzzles, according to well-defined rules, and is best solved using a recursive strategy (Goel & Grafman, 1995a). We used an open-source computer version of ToH retrieved from The Psychological Experiment Building Language (PEBL; http://pebl.sf.net/battery.html; Mueller, 2011, Peirce, 2007). In this computerized version, participants were exposed to ten trials with increasing levels of difficulty. Level of difficulty was indexed by the minimum number of movements required to complete the trial. Participants were instructed to solve the trials mentally before starting to move the disks. The final score in this task was the length of the most challenging problem each participant could solve without mistakes. This score reflected the ability to generate and maintain recursive movement sequences before initiating the motor program necessary to solve the task (Halford et al., 1998, Halford et al., 2010).

#### 3.1.5 Melodic Memory Task (MMT)

This task was an authorized adaptation of the MMT to the Psychopy program (Peirce, 2007), originally part of the Goldsmiths Musical Sophistication Index battery (Gold-MSI) (Mullensiefen et al., 2014). The goal of this task is to assess participants’ memory for short melodies. Participants were asked to listen to pairs of short melodies (containing between 10 and 17 notes) and to indicate whether the two melodies had an identical pitch interval structure or not (by selecting “same” or different”). Participants also rated the confidence of their judgment on a 3-point scale (“I’m totally sure”, “I think so”, “I’m guessing”). In “same” trials, the second melody had the same pitch interval structure as the first one, but was transposed by a semitone or by a fifth. In “different” trials, in addition to being transposed, the second melody was modified by changing two notes by an interval varying between 1 and 4 semitones (for details, see Mullensiefen et al., 2014). The task was composed of 13 trials, including 2 initial training trials, and had a total duration of around 10 min. Accuracy was calculated as d’ scores.

#### 3.1.6 Procedure

All participants performed ART, AIT, VRT, VIT, ToH and MMT. The procedure was divided in two blocks of 50 min each: the ‘auditory block’ - comprised of ART, AIT, and MMT - and the ‘non-auditory block’ - comprised of VRT, VIT and ToH. Each participant executed the two blocks with a break of 10 min in between. The order of the blocks was counter-balanced: 20 participants started the procedure with the ‘auditory block’ and 20 participants started with the ‘non-auditory block’. The order of tasks within each block was also counter-balanced: 20 participants started both blocks with recursive tasks first (e.g., ART-MMT-AIT-break-VRT-ToH-VIT) and 20 participants started each block with iterative tasks first (e.g., AIT-MMT-ART, VIT-ToH-VRT). ToH and MMT were always performed in the middle of their blocks.

All tasks were executed in the same room and computer screen, and auditory stimuli were delivered through headphones. The procedure had a total duration of 2 h.

#### 3.1.7 Analysis

First, we standardized the data by doing *z*-transformations. Then, we performed exploratory correlational analysis using Spearman correlations, and stepwise correlational analyses to determine the best model fit to predict both ART and AIT performance. We used the stepAIC function from the R library MASS (Venables & Ripley, 2002). Finally, we submitted all variables to a Principal Component Analysis (PCA) with Varimax rotation. The PCA was performed with SPSS 22.

### 3.2 Results

#### 3.2.1 Replication of Experiment 1

In Experiment 2, we did not enforce strict inclusion criteria regarding music training since we were interested in parametric differences between participants. Thus, the sample included participants with a wide range of musical training (from 0 to 16 years). However, when we applied (post hoc) the same criteria to classify musicians and non-musicians as in Experiment 1, we obtained similar results: Musicians (8 years or more of musical training, N = 7) were more accurate in ART (*M* = 96%, *SD* = 8%) than non-musicians (less than 2 years of musical training, N = 23, *M* = 76%, *SD* = 22%) (Mann-Whitney U: *z* = −2.53, *p* = 0.012). We also found that musicians were more accurate in AIT (*M* = 90%, *SD* = 14%) than non-musicians (*M* = 73%, *SD* = 20%) (Mann-Whitney U: *z* = -2.00, *p* = 0.05). This confirms that the effect of musical expertise is present for both the recursive and iterative tasks.

#### 3.2.2 Correlational analyses

We z-transformed all variables (raw data is presented in Table 1) to homogenize the scales, and performed exploratory correlation analyses. We found that performance in both ART and AIT was correlated with the number of years of music training (*r_s_* = 0.38, *p* = 0.01; *r_s_* = 0.34, *p* = 0.04, respectively), with performance in the Melodic Memory (*r_s_* = 0.36, *p* = 0.02; *r_s_* = 0.33, *p* = 0.04, respectively) and Visual Recursion tasks (*r_s_* = 0.37, *p* = 0.02; *r_s_* = 0.32, *p* = 0.05, respectively) (see Table 2).

To assess whether there were specific differences between ART and AIT, we performed two stepwise regression analyses, with both AIT and ART as dependent variables. ART was best predicted by a model including AIT (*b* = 0.69, *t*(35) = 8.8, *p* < 0.001), VRT (*b* = 0.15, *t*(35) = 2.1, *p* = 0.04), ToH (*b* = 0.15, *t*(35) = 1.5, *p* = 0.14) and Music training (*b* = 0.13, *t*(35) = 1.4, *p* = 0.18) (model AIC = −40.84; significance tests conducted on the predictors entered in the same order as above, using Type 1 sums of squares). In the inverse analysis, AIT was best predicted by a model including only ART (*b* = 0.8, *F*(1,35) = 67.9, *p* < 0.001).

#### 3.2.3 Principal component analysis

We then submitted all variables to a Principal Component Analysis. The analysis clustered the data into two Principal Components (*KMO* = 0.68, *Bartlett’s Test for Sphericity*:  χ^2^(21)= 75.6, *p* < 0.001): the first explaining 41.6% of the variance and the second 15.8%. Results with Varimax rotation are depicted in Table 3.

With the exception of the Tower of Hanoi, all tasks had a loading coefficient higher than 0.35 on Principal Component 1 (PC1), which explained the most variance and may reflect general cognitive capacity. The tasks with higher loading in this component were AIT, ART, as well as music training, which hints of some predominance of auditory perception. On the other hand, Principal Component 2 (PC2) was dominated by ToH, which is known to be a recursive task (Halford et al., 1998, Halford et al., 2010). Both VRT and ART had higher loadings on this component, suggesting that PC2 might have some specificity for recursive tasks. Interestingly, MMT also had a high loading on this component, but not VIT, AIT or musical training. This suggest that in addition to general auditory processing, MMT might also require complex hierarchical processing.

### 3.3 Discussion

In these experiments, we sought to investigate whether the Auditory Recursion Task tapped into cognitive constructs specific to recursion. We did this by testing each participant with a battery of tests including ART, AIT, two non-auditory recursion tasks (VRT and ToH), a non-auditory non-recursive task (VIT), and a melodic memory task (MMT); we then performed several correlational analyses.

The first and core finding of our experiments was a confirmation that humans can represent recursion in the auditory domain. When we tested participants with different levels of musical expertise (from 0 to 16 years of music training) global performance in ART rose to 82%, in more musically-trained participants. We also confirmed that musical training was a major predictor of performance in both VRT and ART, as was the ability to detect changes in melodic contour (MMT). Notably, performance was higher in Experiment 2 than in Experiment 1, both for musicians and non-musicians, and both for ART and AIT. This might have been caused by task order effects, since in Experiment 2 participants performed six tasks instead of one. Consistent with this interpretation, when participants started the procedure with the ‘visual block’ (VRT, VIT and ToH), performance was on average 82% in AIT and 86% in ART. When participants started the procedure with the ‘auditory block’ (ART, AIT and MMT), performance was on average 74% in AIT and 78% in ART.

The second finding was that, when we controlled for the variance explained by a control auditory task – AIT – in all respects identical to ART but without recursion, we found that ART was specifically predicted by VRT and ToH (two non-auditory recursive tasks) (Halford et al., 1998, Halford et al., 2010, Martins et al., 2015). This result was confirmed by a PCA. The latter analysis yielded two major components, one relating with general cognitive capacity, strongly influenced by musical training and both AIT and ART, and another component, PC2, explaining 16% of the variance, which was dominated by the three recursive tasks. However, performance on the MMT task also loaded strongly on this putative ‘domain-general’ recursive component. This finding is intriguing and invites the speculation that perhaps the explicit processing of musical structure in melodies invokes some sort of domain-general hierarchical or recursive processing, as suggested by Lerdahl & Jackendoff (Jackendoff and Lerdahl, 2006, Lerdahl and Jackendoff, 1983, Lerdahl and Jackendoff, 1996). Alternatively, the component (PC2) comprising VRT, ART, ToH and MMT could be accounted by general intelligence in some way. However, we think that PC1 is more likely than PC2 to represent ‘general capacity’, given that all variables (VRT, VIT, ART, AIT, MMT and Music training) had moderate loadings on this component with the exception of ToH. Furthermore, memory for musical phrases (as tapped by MMT) has been shown to be somewhat independent of general intelligence (Pring et al., 2008, Steinke et al., 1997); and IQ seems to be also weakly correlated with VRT (Martins, Fischmeister, et al., 2014) and ToH (Bishop et al., 2001, Byrnes and Spitz, 1979, Goel and Grafman, 1995b), even though IQ might explain a small proportion of variance in these tasks (Martins, 2012, Zook et al., 2004). Taken together, previous research and current findings make PC2 unlikely to reflect a ‘general intelligence’ component independent of PC1.

Finally, when we accounted for the shared variance with ART, we did not find specific correlations between the AIT and other cognitive measures, including with VIT. Instead, this task was strongly related with other auditory measures. This suggests that the auditory processing of simple iteration relies more on modality-specific resources that do not generalize across domains.

## 4 General discussion

In this article we introduced two novel tasks – AIT and ART – designed to test participants’ ability to represent iterative and recursive processes in the auditory domain. Through a series of two experiments, we have shown that human adults can process and represent the recursive structure underlying the generation of music fractals, and can use this information to discriminate between well-formed hierarchies and multiple categories of foils.

In the first experiment, we found that performance was consistent with the induction of an abstract rule: participants could correctly reject different foil categories and the increase in accuracy across trials was highly suggestive of a learning effect. Second, we found that musical expertise had a significant impact on the ability to extract a recursive rule from sequences of tones. While musicians displayed an accuracy level similar to levels previously shown by the general population in a *visual* recursion task (Martins, 2012), non-musicians had a significantly lower score (71%). This specific difficulty in the auditory domain could be caused by proximal factors, involving attention to or perception of tone sequences (Bigand and Poulin-Charronnat, 2006, McDermott et al., 2010, Tervaniemi et al., 2005) and/or distal factors, more related to the extraction of abstract principles from musical stimuli (Besson and Faïta, 1995, Besson et al., 1994, Cook, 1987, Tillmann, 2012, Tillmann et al., 1988). Performance could also be constrained by auditory memory limitations in non-musicians (Cohen et al., 2011). Interestingly, non-musicians’ performance was already low in AIT, a task simpler than ART, in which participants only had to discriminate the correct directionality (ascending or descending) of a sequence of three tones. This suggests that the difficulty of non-musicians was partially mediated by proximal factors related with the ability to perceive melodic structure even in very simple stimuli (Bigand & Poulin-Charronnat, 2006) or to discriminate between different pitch intervals (McDermott et al., 2010), possibly related to a difficulty directing attention to the relevant acoustic parameters (Tervaniemi et al., 2005).

In the second experiment, we applied a larger test battery to a new set of participants; the battery included not only auditory recursive and iterative tasks, but also visual recursion, visual iteration, melodic memory and Tower of Hanoi (a visuo-motor recursive task). The results of this experiment strongly suggest that ART correlates well with other recursive abilities, but that it also depends on general capacities to process auditory stimuli. This finding is consistent with previous research showing that global-to-local interference effects in the processing of hierarchies is significantly correlated between visual and music modalities (Bouvet et al., 2011). Here we have extended this result to the representation of recursion.

### 4.1 Is recursion domain-general?

Recursion as a cognitive capacity allows the generation of multiple hierarchical levels with a single rule (Fitch, 2010, Hulst, 2010, Martins, 2012). This makes recursion especially useful in the processing and generation of complex hierarchies, with a specific potential to build hierarchies of very large depth (Hauser et al., 2002, Koike and Yoshihara, 1993, Schiemenz, 2002). Recursion has been claimed to be present in many domains (Alegre and Gordon, 1996, Arsenijević and Wolfram, 2010, Busi et al., 2009, Chomsky, 1995, de Vries et al., 2011, Eglash, 1997, Eglash, 1998, Eisenberg, 2008, Fitch, 2010, Fitch et al., 2005, Hauser et al., 2002, Hulst, 2010, Hunyady, 2010, Jackendoff and Lerdahl, 2006, Karlsson, 2010, Koike and Yoshihara, 1993, Komara, 2011, Latek et al., 2009, Laury and Ono, 2010, Levinson, 2013, Martins et al., 2015, Odifreddi, 1999, Roeper, 2011, Schiemenz, 2002, Schreuder et al., 2009, Wagner, 2010). However, it seems to be most frequently (and universally) used in the domain of language (Arsenijević and Wolfram, 2010, Goldberg, 2003), and available in this domain from an early age (Alegre and Gordon, 1996, Roeper, 2011). The latter facts have led some theorists to postulate that recursion might have evolved in the domain of language first, only later becoming available to other domains, with the caveat that its use would be dependent on language resources (Fitch et al., 2005, Hauser et al., 2002).

In previous research, we have shown that recursion is available in the visuo-spatial domain (Martins, 2012) and that its use does not require language resources, as evidenced by interference (Martins et al., 2015), developmental (Martins, Laaha, et al., 2014) and brain imaging studies (Martins, Fischmeister, et al., 2014). But even if classic language resources are not necessary to process recursion in vision, it remained an open question whether recursion is domain-specific or domain-general. The data presented here strongly suggest the latter. In this study we show for the first time not only that recursion can be processed in the non-linguistic auditory domain, but that the capacity to form recursive representations strongly correlates with the same ability in the visual and action sequencing domains. Notably, this domain generality was *not* present for the control Auditory Iteration Task, which did not correlate with the Visual Iteration Task. Thus, despite the obvious influence of domain-specific constraints in the processing of auditory information, probably located at the interface between the sensory level and higher cognition (Cohen et al., 2011, Tervaniemi et al., 2005), the capacity to build recursive representations might be instantiated by a more general and abstract code, thus constituting a core computational capacity available to many different domains of cognition.

In the future we intend to better situate the current results in the broader debate of language evolution, by incorporating specific linguistic recursion tasks into our test battery. We will also perform neuroimaging experiments with these auditory tasks and compare the results with the networks active during visual recursion. This research will increase our understanding of this potentially human-specific capacity, and further specify its role in our unique cognitive architecture.

## Figures and Tables

**Table 1:** Descriptive data for all the measures included in the battery. ART (Auditory Recursion Task), VRT (Visual Recursion Task), AIT (Auditory Iteration Task), VIT (Visual Iteration Task), ToH (Tower of Hanoi), MMT (Melodic Memory Task), and Musical training.

	N	Minimum	Maximum	Mean	SD
ART	40	0.42	1.00	0.82	0.20
VRT	40	0.52	1.00	0.89	0.12
AIT	40	0.33	1.00	0.78	0.19
VIT	40	0.70	1.00	0.88	0.09
ToH	40	3.00	7.00	5.82	1.06
MMT (d′)	40	−0.84	3.46	1.07	0.97
Music training (years)	40	0.00	16.00	3.27	4.02

**Table 2:** Correlations between AIT (Auditory Iteration Task), ART (Auditory Recursion Task) and the other variables. MMT (Melodic Memory Task), MTrain (Music training), ToH (Tower of Hanoi), VIT (Visual Iteration Task), and VRT (Visual Recursion Task). ^**^p < 0.01 ^*^p < 0.05. Correlation coefficients are Spearman’s rho.

		AIT	MMT	MTrain	ToH	VIT	VRT
AIT	*r_s_*		0.33^*^	0.34^*^	−0.00	0.17	0.32^*^
ART	*r_s_*	0.83^**^	0.36^*^	0.38^*^	0.13	0.28	0.37^*^

**Table 3:** Principal component analysis. AIT (Auditory Iteration Task), ART (Auditory Recursion Task), Music training, VIT (Visual Iteration Task), ToH (Tower of Hanoi), MMT (Melodic Memory Task) and VRT (Visual Recursion Task).

	Component	
	1	2
AIT	0.769	
ART	0.753	0.410
Music training (years)	0.732	
VIT	0.543	
ToH		0.838
MMT (d′)	0.356	0.631
VRT	0.386	0.624

## Appendix A Supplementary material

Supplementary Material I

Supplementary Material II

Supplementary Material III

Supplementary Material IV
