# Supplementary material for: Cognitive representation of “musical fractals”: Processing hierarchy and recursion in the auditory domain
Source: Cognition. 2017 Apr;161:31–45. doi: 10.1016/j.cognition.2017.01.001 (PMC5348576; doi:10.1016/j.cognition.2017.01.001)

COGBIO
Department of Cognitive Biology

universität wien

Experiment Information

Experiment information

- This is an experiment about auditory perception. It will be composed of 24 trials.
- On each trial, you will be presented with four sounds composed of sequences of tones. Listen very carefully to these sequences of tones.

Trial example

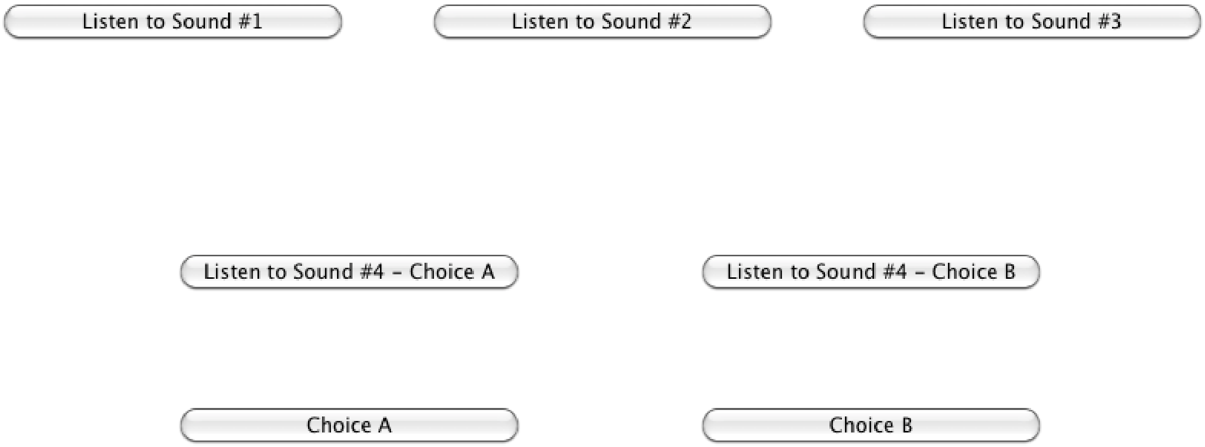

Several buttons will be displayed on the screen. You can left-click these buttons in order to listen to the sounds.

Trial example

Listen to Sound #1

Listen to Sound #2

Listen to Sound #3

Listen to Sound #4 – Choice A

Listen to Sound #4 – Choice B

Choice A

Choice B

This is Sound #1!

Trial example

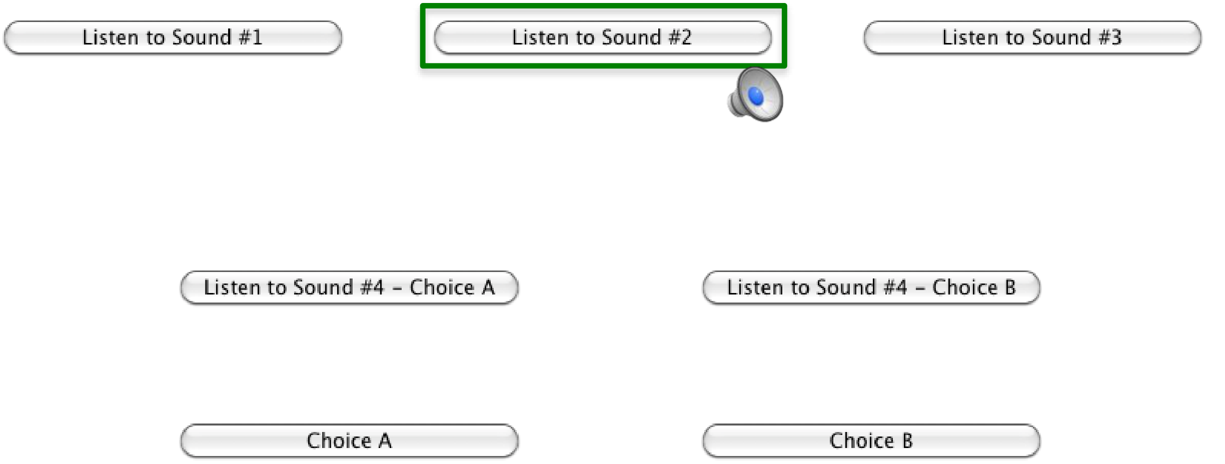

This is Sound #2!

Trial example

Listen to Sound #1

Listen to Sound #2

Listen to Sound #3

Listen to Sound #4 – Choice A

Listen to Sound #4 – Choice B

Choice A

Choice B

This is Sound #3!

Trial example

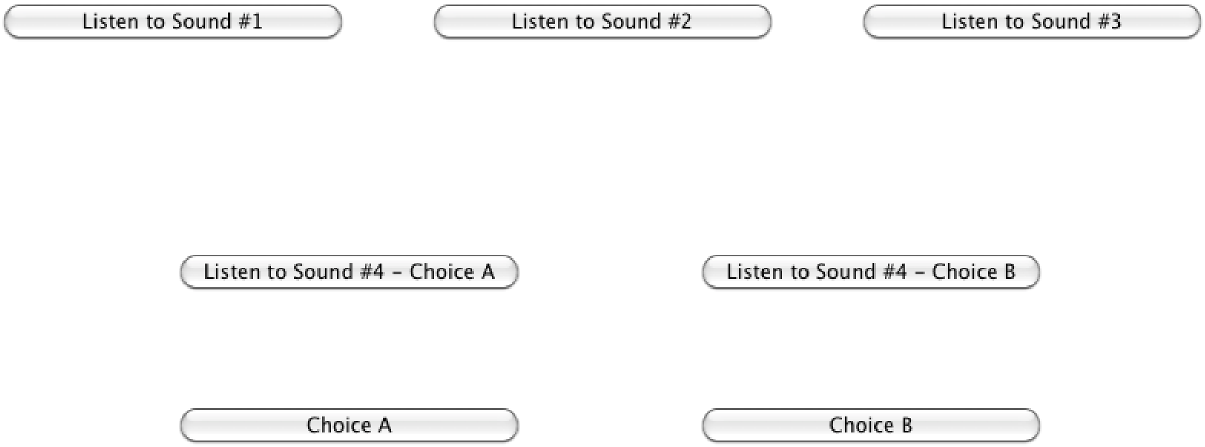

After listening to the first three sounds,
try to imagine how 'Sound #4' would be like.
Try to play it in your mind.
If needed, listen to the previous sounds again.

Trial example

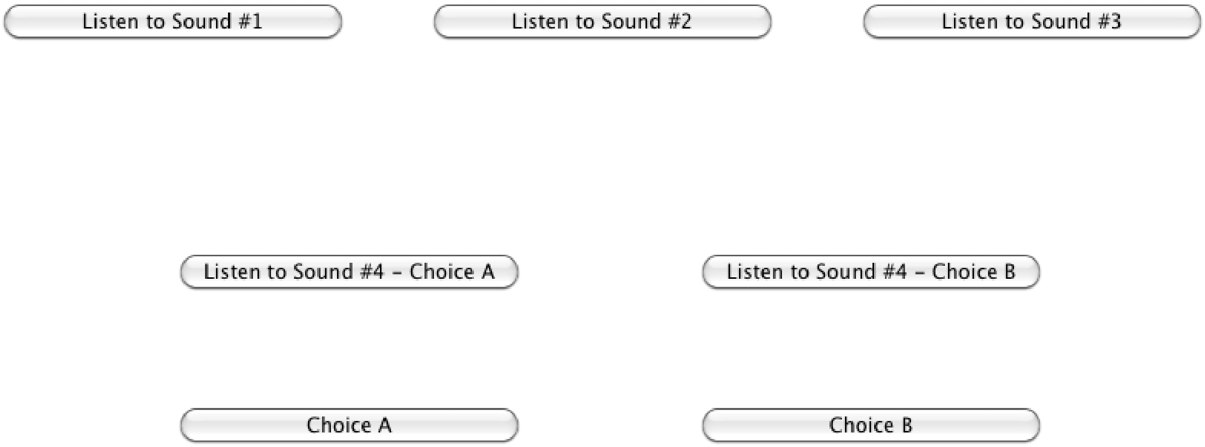

Now listen to ‘Sound #4 – Choice A’ and ‘Sound #4 – Choice B’.

Trial example

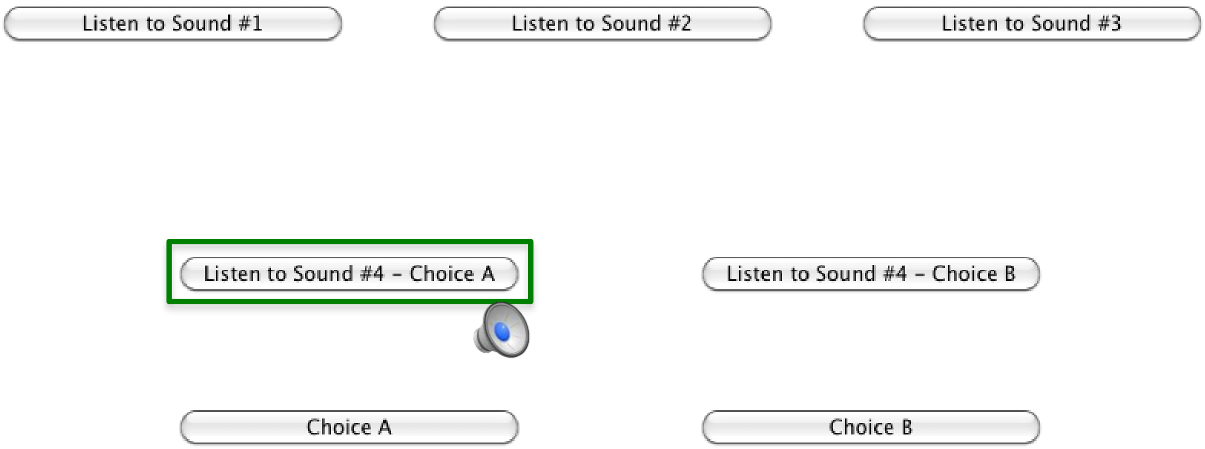

Sound #4 – Choice A!

Trial example

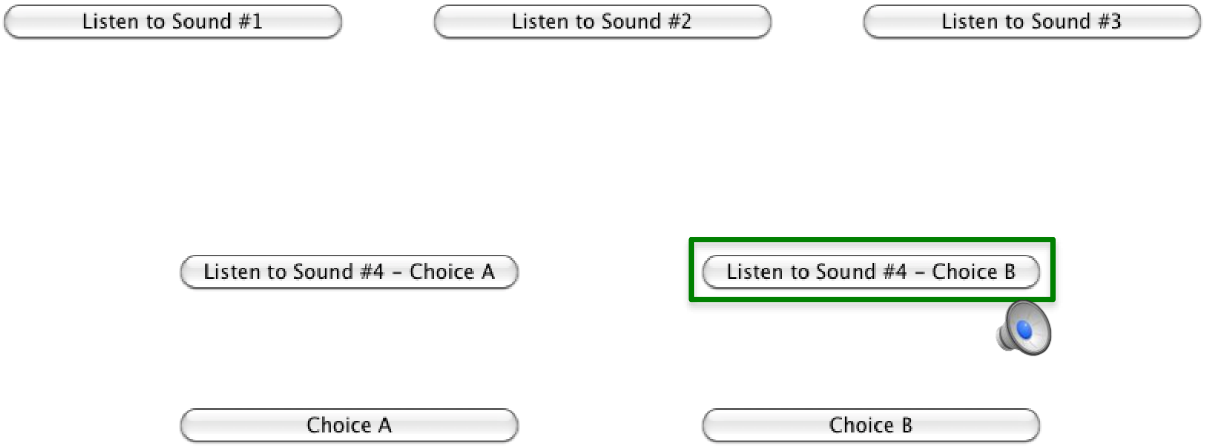

Sound #4 – Choice B!

Trial example

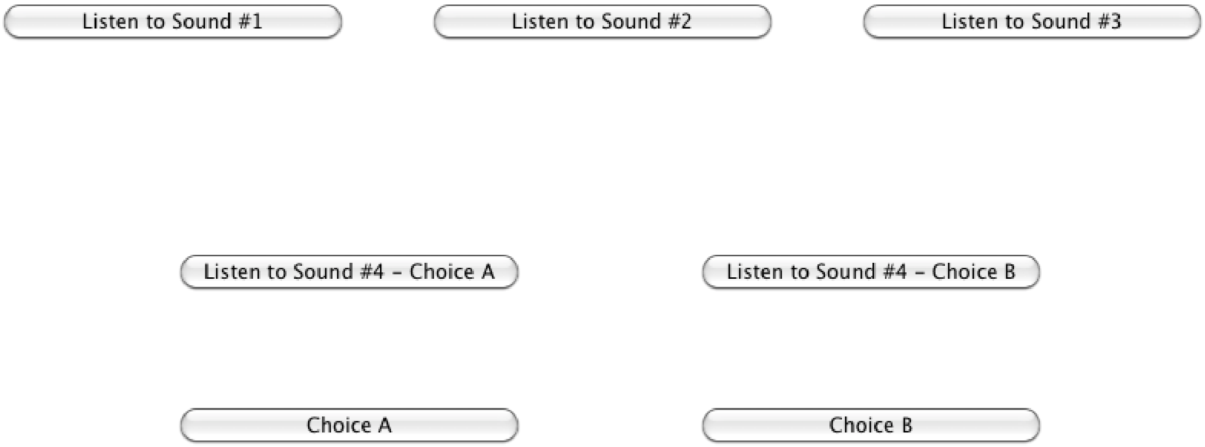

Which of the two sounds correctly continues Sound #1, 2 and 3? Which is the correct Sound #4? Choice A or Choice B?

Trial example

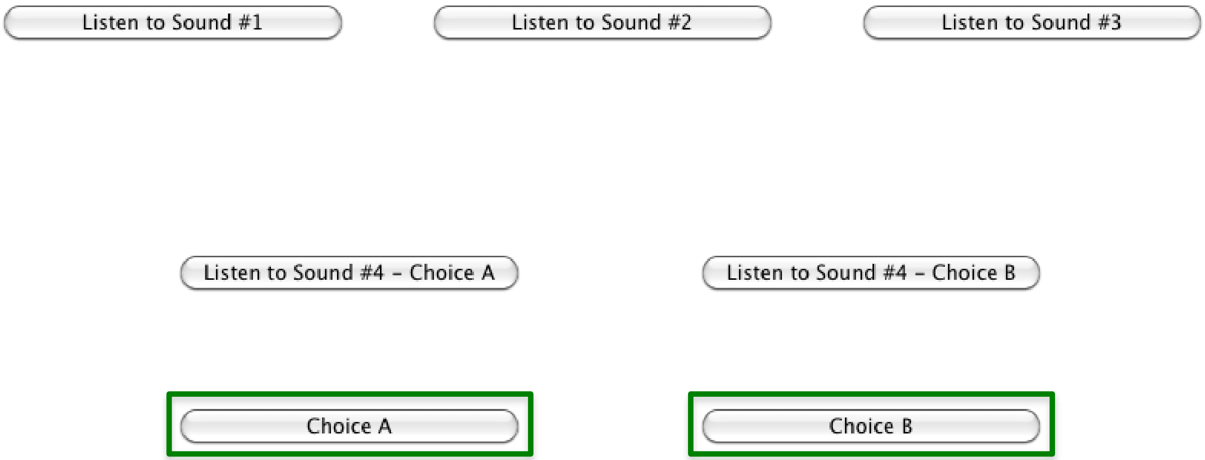

Present your choice by left-clicking the corresponding button. Listen to both choices very carefully before making your decision, and try not to answer until you feel confident about your choice.

Trial example

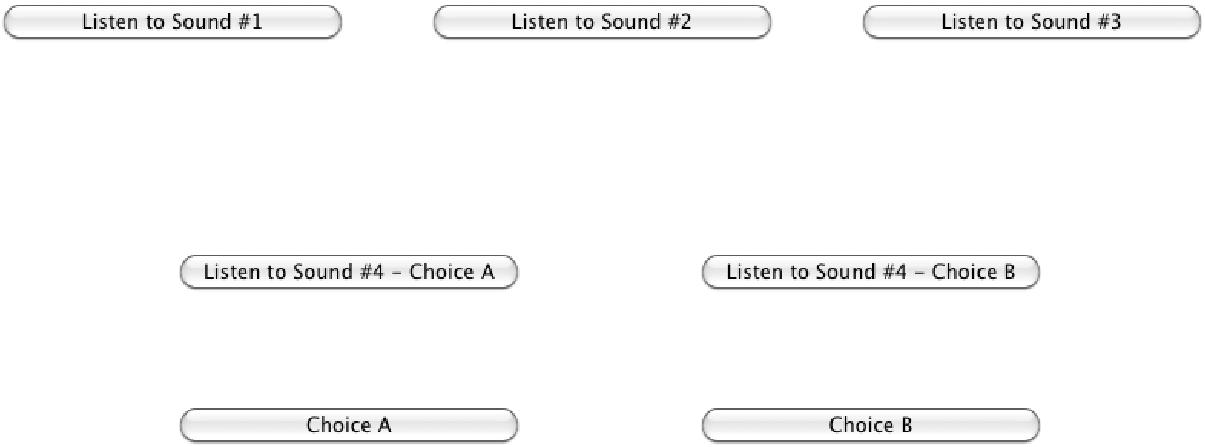

Choice A and Choice B are always different.
If it is not clear how they are different, or which one is the correct answer, please repeat the listening.
We encourage you to listen to all sounds as many times as you need.

Trial example

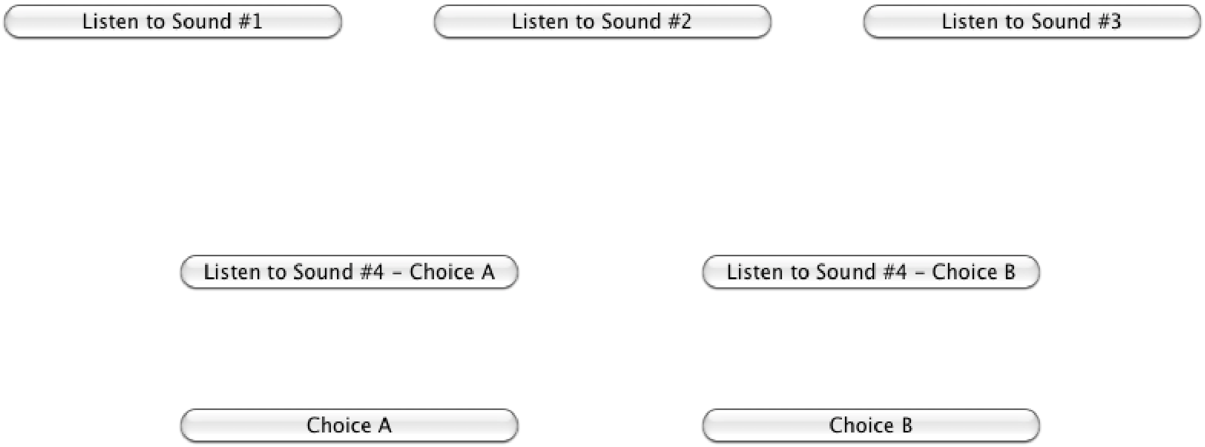

At the beginning of each trial, sounds #1, #2, #3 will be played automatically. After the automatic play of these sounds, you can then click the buttons to repeat the listening of each sound.

Experiment information

- Before the experiment, you will be given a training session, composed of 24 trials.

- In this training you will be presented with a sound #1, which is composed of a sequence of tones. You will be asked to determine which of two alternatives (A or B) correctly continues sound #1.

Any questions?

Ready to go?

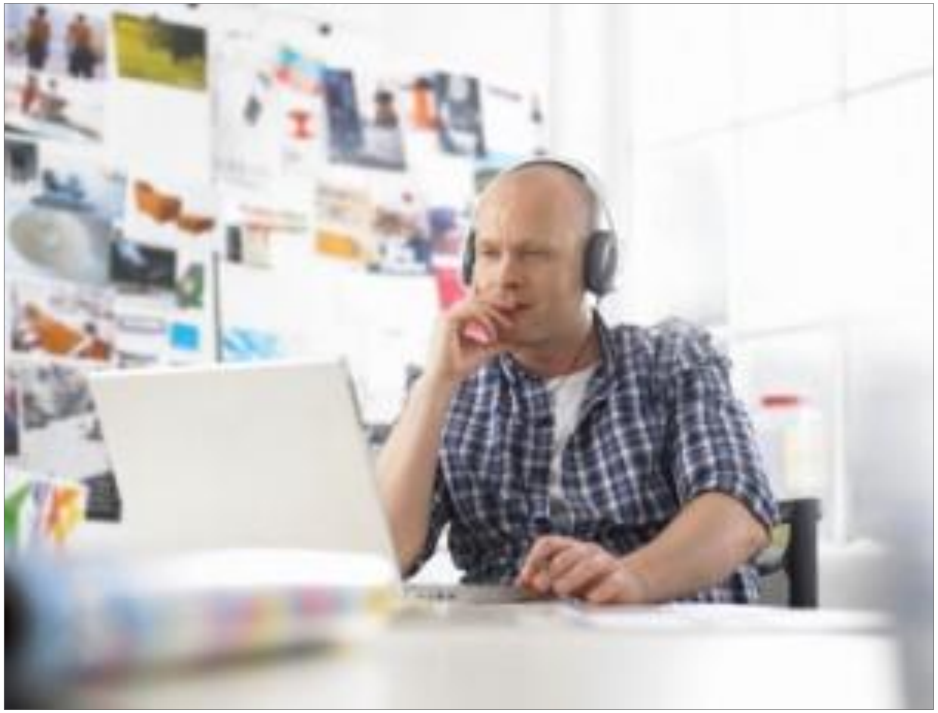

Supplement: Supplementary Material IV [file mmc4.pdf]
